# Correlation of Elemental Transfer, Bioactive Compounds and Antioxidant Activity on *Lactuca sativa* L. Grown in Soil with Functionalized CNT and HMs

**DOI:** 10.3390/metabo13121171

**Published:** 2023-11-24

**Authors:** Maria-Loredana Soran, Ildiko Lung, Adina Stegarescu, Otilia Culicov, Ocsana Opriș, Pavel Nekhoroshkov, Dorina Podar

**Affiliations:** 1 National Institute for Research and Development of Isotopic and Molecular Technologies, 67-103 Donat, 400293 Cluj-Napoca, Romania; loredana.soran@itim-cj.ro (M.-L.S.); ildiko.lung@itim-cj.ro (I.L.); adina.stegarescu@itim-cj.ro (A.S.); ocsana.opris@itim-cj.ro (O.O.); 2 Joint Institute for Nuclear Research, 6 Joliot-Curie, 141980 Dubna, Russia; pavel@nf.jinr.ru; 3 National Institute for Research and Development in Electrical Engineering ICPE-CA, 313 Splaiul Unirii, 030138 Bucharest, Romania; 4 Department of Molecular Biology and Biotechnology, Faculty of Biology and Geology, Babeș-Bolyai University, 1 Kogălniceanu St., 400084 Cluj-Napoca, Romania; dorina.podar@ubbcluj.ro

**Keywords:** lettuce, heavy metals, functionalized carbon nanotubes, assimilatory pigments, polyphenols, DPPH, elemental content, neutron activation analysis (NAA)

## Abstract

While heavy metals (HM) have been considered in recent decades to be the most common and problematic pollutants, the expansion of the list of pollutants due to the active use of carbon nanotubes (CNT) raises new questions about the benefit and harm of HM released to nature individually or fixed on CNT walls. A pot experiment was conducted to compare the effect of two classes of potential pollutants—metal salts of Pb, Mn, Cu, Zn, Cd, and Ni; and functionalized CNTs with COOH, MnO_2_, Fe_3_O_4_, and MnO_2_-Fe_3_O_4_—applied in soil, on the elemental transfer, the bioactive compounds accumulation, and the antioxidant activity in lettuce. While CNTs mainly increased the elemental transfer from soil to leaves, HM salts strongly obstructed it. In the presence of CNTs, the antioxidant activity in lettuce leaves correlated with the transfer of elements from soil to root and from root to leaves. The excess of HMs in soil induced a greater variation of the polyphenols quantity and antioxidant activity than the excess of CNTs. It might be assumed that lettuce perceived HMs as a more aggressive stressor than CNTs and more strongly activated the defense mechanism, showing the reduction of the element transfer and enhancing of total polyphenol production and antioxidant activity.

## 1. Introduction

Lettuce (*Lactuca sativa*) occupies one of the leading places among the most widely consumed leafy vegetables worldwide. However, lettuce can easily incur different abiotic stress factors such as water deficit, pathogens, salinity, soil contamination with heavy metals (HMs), and the presence of pharmaceutical products in soil [1,2,3,4,5,6]. Lettuce is considered a valuable model plant species for phytotoxicity studies that include both single and multi-contaminants [7]. Recently, the risks for soil contamination with carbon nanotubes (CNTs) and its potential impact on plants has also attracted the attention of the scientific community [8]. 

For several decades, the continuous spread of organic and inorganic fertilizers, as well as the application of sludge from wastewater treatment plants as fertilizers, has progressively led to the accumulation of the metals in soils. The soil pollution with heavy metals is persistent due to the long half-time persistence of the contaminants in the environment and can be harmful along the whole food chain [9,10]. 

The unique properties of CNTs such as high thermal and electrical conductivity, elasticity, tensile strength, flexibility, etc., are expanding their uses in practically all industries and also in agriculture [11]. CNTs functionalized with metal oxide nanoparticles form a new class of hybrid nanomaterials, which, in addition to beneficial properties gained due to the interactions between CNTs and attached metal oxide nanoparticles [12], may potentially lead to additional harm to the environment. 

The use of nanoparticles, as well as the use of heavy metals, has both negative and positive effects on plants that depend upon the plant species and the type and concentration of nanoparticles used [13]. CNT might be used as fertilizers to enhance plant growth, as pesticides for disease management, or even as sensors to monitor plant health and soil quality [14]. However, some studies have shown that CNTs can lead to phytotoxic effects: decreased plant growth, increased generation of reactive oxygen species, or decreased cell dry weight [15,16].

It is important to note that the majority of the studies concerning the effects of HM and CNT on plants are performed on hydroponic cultures; therefore, it is quite bold to extrapolate the results to real plant growth conditions in soil. The use of soil as substrate influences both plant–HM and plant–CNT interactions via different aspects: root architecture (differs in soil vs. in hydroponics), soil pH modified by the addition of HM, CNT behavior, which differs in the presence of ions from the soil solution than in hydroponics, and both HM and CNT, which interact with ions and organic matter from the soil, probably reducing the mineral elements bioavailability for the plants [17]. Furthermore, bacterial activity within the rhizosphere is affected as well. The use of CNT functionalized with heavy metals induces even more uncertainty to the fate of minerals within the soil and their uptake by plants. 

For a long time, HMs were considered the most common and problematic soil pollutants; thus, many studies have focused on their impact on plants. The expansion of the list of pollutants in recent years, due to appearance and applications of CNTs and CNTs functionalized with HMs, raises new questions concerning the benefit and harm of HM used individually or fixed on CNT walls. The majority of studies focused on the impact of HM and CNT on plants, paying attention to plant growth, roots, and leaves elongation [18], germination parameters [19], and foliar degeneration, follow the uptake of target pollutant to plant [20], the content of bioactive compounds, and the antioxidant capacity of the plants [21], but practically ignore the modification of the multi-elemental patterns of soil, root, and plant during the experiment. Studies focused on individual stressors have shown that the application of CNTs to soil changed the availability of some HMs and nutrients in soil, which may be both beneficial and harmful to plants [22]. The spectra of elements investigated have to be enlarged since the use of HMs and CNTs in many vital sectors may lead to individual or simultaneous inclusion to the soil–plant system. 

The aim of this study was to evaluate and compare the impact of HMs (Pb, Mn, Cu, Zn, Cd, and Ni) and CNTs functionalized with oxides of HMs (MnO_2_, Fe_3_O_4_, MnO_2_-Fe_3_O_4_) on the soil–lettuce system, including the mineral elements uptake and transfer in the plant, the variation of the content of bioactive compounds, and the antioxidant activity of the lettuce. Correlation between multi-elemental transfer from soil to plant, the content of bioactive compounds, and the antioxidant activity of lettuce grown in soil substrate with heavy metal salts and functionalized carbon nanotubes was also assessed. 

## 2. Materials and Methods

### 2.1. Plant Growth Conditions

Seeds of the *Lactuca sativa* variety Attraction(S.C. Agrosem Impex S.R.L., Târgu-Mureș, Romania) were cultivated in a garden substrate with active humus and fertilizer for six weeks, produced by AGRO CS Slovakia, a. s. Nám. Republiky 5, 98401 Lucenec, SK, being distributed by AGRO CS Romania S.R.L. 

Two sets of experimental treatments were designed: one set including the addition of CNT-COOH, CNT-Fe_3_O_4_, CNT-MnO_2_, and CNT-Fe_3_O_4_-MnO_2_ in soil in a concentration of 2.33 mg kg^−1^; and a second set, where soil was exposed to heavy metal salts: copper(II) chloride di-hydrate (CuCl_2_·2H_2_O, 100 mg kg^−1^), cadmium acetate dihydrate (Cd(CH_3_COO)_2_·2H_2_O, 3 mg kg^−1^), zinc acetate dihydrate (Zn(CH_3_COO)_2_·2H_2_O, 300 mg kg^−1^), manganese(II) chloride tetrahydrate (MnCl_2_·4H_2_O, 1500 mg kg^−1^), nickel chloride (NiCl_2_, 75 mg kg^−1^), and lead(II) sulfate (PbSO_4_, 50 mg kg^−1^). Commercial soil without the addition of HMs or CNTs was used as a control. 

The synthesis and characterization of the nanoparticles used in this study were reported in the previous papers [23,24,25], as well as the cultivation conditions [26,27].

### 2.2. Plant and Soil Analysis after Harvesting

#### 2.2.1. Determination of Chlorophylls and Total Carotenoids Concentration

The pigments extraction method was performed as previously described in [27]. Lichtenthaler’s calculation formulae were used for the determination of the pigments concentration (chlorophyll a and chlorophyll b, as well as carotenoids) [28].

#### 2.2.2. Total Polyphenols Evaluation

The alcoholic extracts were obtained from fresh lettuce leaves with 60% ethanol in a ratio of 1:40 (*w*/*w*) via sonication for 30 min at room temperature, using an Elma Transsonic T ultrasonic bath. The obtained mixture was centrifuged for 10 min at 7000 rpm and the supernatant was stored at 4 °C until analysis. The total polyphenols content was determined via the Folin–Ciocalteu method described by Ivanova et al. [29]

#### 2.2.3. Antioxidant Capacity Determination via DPPH Method

The antioxidant capacity of the hydroalcoholic extracts was assessed via a slightly modified procedure by Brand-Williams et al. [30] and described in Soran et al. [27].

#### 2.2.4. Multi-Elemental Investigation of Lettuce and Soil Substrate via NAA

Up to 0.3 g of dry biological material (root and leaves individually) and 0.1 g of cultivation substrate were weighed on a digital microbalance and sealed in high-purity polyethylene and aluminum foil for short and long irradiation, respectively. To determine short-lived isotopes, the samples were irradiated for 3 min under a thermal neutron fluency rate of approximately 1.6 × 10^13^ n cm^−2^ s^−1^. Immediately after irradiation, the short-lived radionuclides were determined from gamma spectra measured for 15 min. Long-term irradiation was performed in a cadmium-screened channel for 3 days under a resonance neutron fluency rate of approximately 3.31 × 10^12^ n cm^−2^ s^−1^. Four days after irradiation, the samples were repacked, and gamma spectra were measured using high-purity germanium detectors. The third measurement was performed at 20–23 days of decay. 

The samples were irradiated within the user program developed at the pulsed fast reactor IBR-2 [I] operated by Joint Institute for Nuclear Research [31].

Twelve elements—Na, K, Fe, Co, Zn, As, Br, Rb, Sr, Sb, Cs, and Sm—were determined for all types of samples (soil, roots, and leaves). Ca was detected both in soil and leaves and Th in roots and soil. Ca was not detected in roots, whereas Th was not detected in leaves.

Quality control of the analysis was performed via use of certified standard trace elements in soil (2709) irradiated in the same conditions as the samples. The measured concentrations and certificate values are in good agreement (Figure S1). The NAA results presented in the paper are expressed on a dry weight basis.

### 2.3. Data Analysis

The values are presented as means of three measurements ± SE (standard error). One-way analysis of variance (ANOVA), followed by Tukey’s test performed with Minitab 17 (Minitab Ltd., Coventry, UK), was used to evaluate the statistically significant differences between groups (*p* < 0.05).

Microsoft Office Excel 2010 (Microsoft, Redmond, WA, USA) was used for the calculations, and Origin 8 (OriginLab Corporation, Northampton, MA, USA) for drawing the graphs. 

For identification of radioactive isotopes and spectrum evaluation, Genie 2000 (version 3.4.1) software was used [32]. For element concentration calculations, the ‘‘Concentration’’ software, version 6.13.3, based on the standard comparative method, produced in JINR, was applied. 

### 2.4. Evaluation of the Elemental Translocation

As a measure of the translocation of mineral elements, the following parameters were calculated based on the concentration measured in dry weight (DW) material (soil, roots, leaves):

$$Bioaccumulation\;factor\;\left(BF\right)=\frac{Element\;content\;in\;root\;\left(mg\;kg^{-1}DW\right)\;}{Element\;content\;in\;soil\;\left(mg\;kg^{-1}DW\right)},$$

$$ioaccumulation\;coefficient\;\left(BC\right)=\frac{Element\;content\;in\;leaves\;\left(mg\;kg^{-1}DW\right)\;}{Element\;content\;in\;soil\;\left(mg\;kg^{-1}DW\right)},$$

$$Translocation\;factor\;\left(TF\right)=\frac{Element\;content\;in\;leaves\;\left(mg\;kg^{-1}DW\right)}{Element\;content\;in\;root\;\left(mg\;kg^{-1}DW\right)}.$$

## 3. Results and Discussion

### 3.1. Elemental Translocation

The variation of the elemental translocation is evaluated in terms of relative variation of BF, BC, and TF values from the control, calculated as the ratio of the value obtained in the presence of the pollutant and the control value.

#### 3.1.1. Uptake of Minerals from Soil

**Figure 1:**
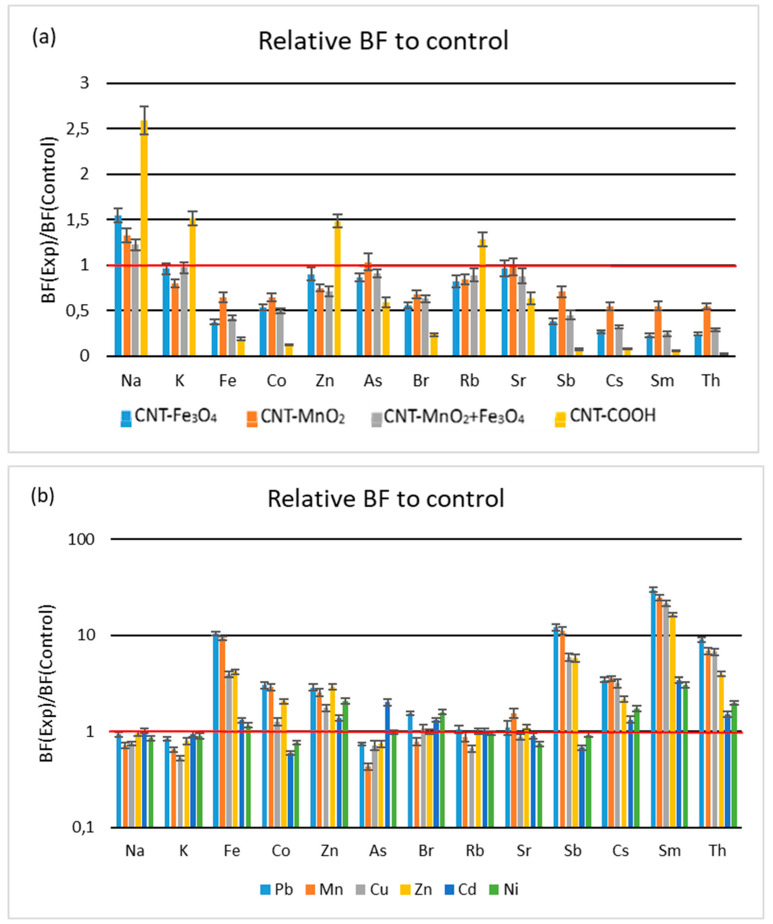
Bioconcentration factors to control plants (**a**) for soils exposed to functionalized CNTs (**b**) for soils exposed to HM salts. Red line corresponds to equal values of BC for control and treatment.

The uptake of mineral elements from soil was assessed through calculation of the bioaccumulation factor, i.e., the ratio of the elemental concentration in roots and in soil. The addition into the soil of all types of CNTs functionalized with HMs leads to a reduced uptake of the elements from soil into the roots, compared to control plants, except for Na, for which the uptake was enhanced (Figure 1). The use of CNTs with carboxylic groups caused an increase in the bioaccumulation factor for Na, K, Zn, and Rb, the highest increase being recorded for Na—more than 2.5 times compared to control plants. The use of CNPs with carboxylic groups led to the strongest suppression of the uptake of the other elements (Fe, Co, As, Br, Sr, Sb, Cs, Sm). It was shown that the binding strengths of different divalent metal ions to the carboxylic acid group differ greatly. For example, Pb^2+^ always exhibits stronger binding than Cd^2+^ though the ionic radius of the latter being smaller [33]. In our study, we found a suppression of the bioaccumulation factor, which could mean a strong fixation of the elements in soil, both for elements with a smaller ionic radius, such as Fe, Co, As, Th, and Sb, and for elements with large ionic radii, such as Br. A similar increase in the bioaccumulation factor values occurs for elements with large ionic radii, which are possibly less related to the COOH groups in the soil, such as Na, K, and Rb, but also for Zn, with an ionic radius comparable to that of Sb. Chen and co-workers also found that various MWCNT soil treatments decreased the BF values of As in corn seedlings in comparison to the control [22].

The effects of HMs in soil on minerals uptake were much stronger than those of using CNTs. The use of CNTs led to a strong decrease in mineral elements uptake compared to control for the majority of the determined elements in the treatments used, in addition to its increase in mineral elements uptake by a maximum of 2.6 times for a few elements. The presence of metal salts in soil induced the increase in the elemental level by up to 30 times for Fe, Sb, Th, and Sm, with only a slight decrease for some elements (Figure 1).

The strongest and systematic increase in bioaccumulation factor was noted for those elements whose transfer was hardly suppressed by the use of CNTs, i.e., for Sm, Th, Sb, Cs, and Fe, and, to a smaller extent, for Co and Br, especially when Pb, Mn, Cu, and Zn were used. On the contrary, uptake of Na, K, and Rb was less affected by HMs loading into soil. 

Studies regarding both foliar and soil-induced stress showed that limiting HMs effectively protects plants from HMs stress [34,35].

#### 3.1.2. Root-to-Leaves Transfer

**Figure 2:**
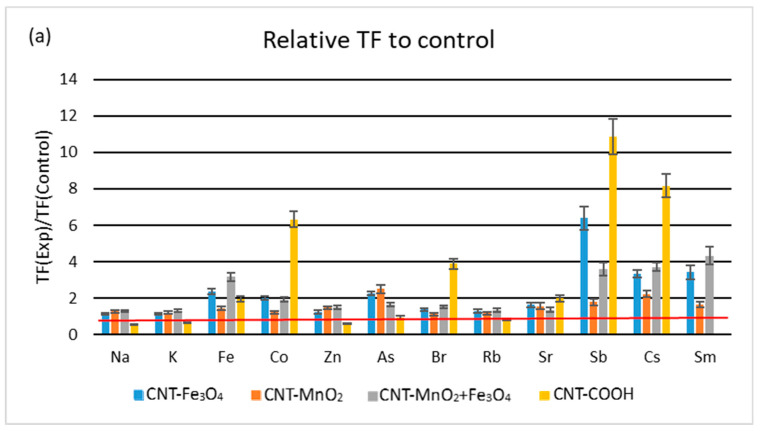

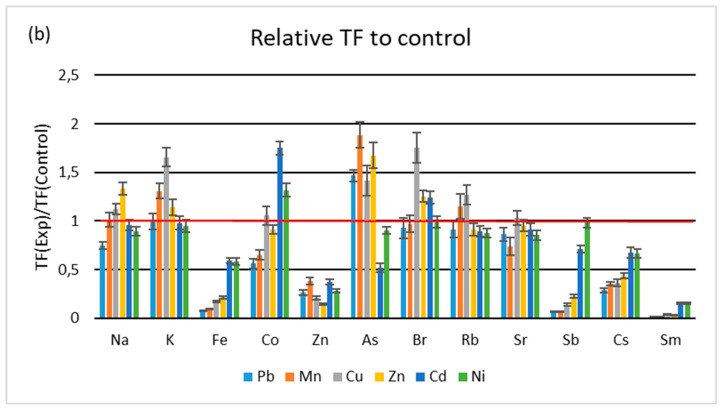
Translocation factor to control plants (**a**) for soils exposed to functionalized CNTs; (**b**) for soils exposed to HM salts. Red line corresponds to equal values of BC for control and treatment.

The elemental transfer from root to leaves was assessed by calculating the translocation factor as the ratio of the elemental concentration in the green, edible part of the lettuce and in the root. The addition of HM-functionalized CNT into the soil stimulated elemental transfer, with the translocation coefficient showing higher values for all elements when lettuce was under the simultaneous impact of CNT and HMs. Among the CNT-HMs, those functionalized with Fe_3_O_4_ increased the transfer to the greatest extent, with elements such as As, Fe, Cs, Sm, and Sb showing translocation coefficients relative to the control ranging from 2 to about 6 (Figure 2a). The transfer of As, Fe, Cs, Sm, and Sb was also enhanced, but to a lesser extent, when Fe_3_O_4_ was associated with CNTs in the presence of MnO_2_. Chen and co-workers [22] noticed also that various MWCNT treatments showed diverse effects on As translocation in corn seedlings subjected to Cd and As stressors, both low and high levels of MWCNTs application significantly increased the TF value of As. 

The addition of CNT-COOH led to a consistent increase in the transfer of Br, Co, Cs, and Sb, the translocation relative to the control showing values ranging between about 4 to more than 10. However, translocation of Na, K, Zn, Rb, and Sm was suppressed (Figure 2a).

While, by addition of functionalized CNT, the transfer reduction is mostly an exception, affecting a small number of elements and appearing only when the CNT-COOH was used, the presence of HM salts in the soil turns this phenomenon into a predominant one; the transfer of most elements is reduced when HM salts are used (Figure 2b). 

The use of Pb salt stimulates As transfer. The translocation of Rb, K and, to a greater extent, As is enhanced by the presence of the Mn salt. The Cu salt slightly enhances the transfer of Co, Sr, and Na, while the translocation of Rb, K, and Br is more strongly enhanced. The use of Zn stimulates the transfer of K, Br, Na, and As. The presence of Cd salt in the substrate amplifies the transfer of Br and Co, while the presence of Ni has the same role only for Co. Fe, Sb, Cs, Zn, and, to the greatest extent, Sm translocation is suppressed by the addition of Ni salt. While relative translocation factor values are up to about 10 in the presence of CNTs, their values are less than 2 when HM salts are used. 

#### 3.1.3. Soil-to-Leaves Transfer

Elemental transfer from soil to leaves is estimated via calculation of the bioaccumulation coefficient as a ratio of elemental concentration in leaves and in soil. 

**Figure 3:**
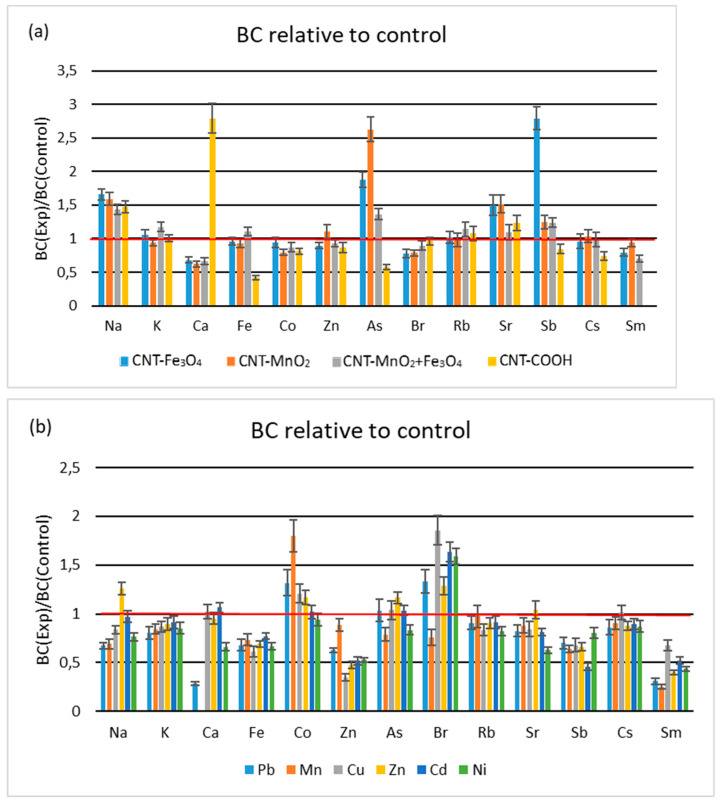
Bioaccumulation coefficient to control plants (**a**) for soils exposed to functionalized CNTs; (**b**) for soils exposed to HM salts. Red line corresponds to equal values of BC for control and treatment.

The use of all functionalized CNTs stimulates the bioaccumulation of Na and, to a lesser extent, Sr in lettuce (Figure 3a). As and Sb translocation are also enhanced by using all HM-functionalized CNTs and reduced when COOH is present in soil. The use of CNT-COOH led to the suppression of all elements transfer from soil to leaves, except for Na, Ca, Rb, and Sr. The addition of all types of functionalized CNTs suppressed the translocation of Co and Br. The least affected by the application of CNT functionalized with HMs was Cs, whose transfer coefficient varied between 0.96 and 1.03. The soil-to-leaves elemental transfer showed extremely increased values for Ca and extremely decreased for Fe when soil was exposed to CNT-COOH. No experimental values for Sm in leaves were obtained in lettuce grown in soil exposed to CNT-COOH; consequently, BC values could not be calculated. 

The elements whose transfer is most stimulated using HMs are Co and Br (Figure 3b). The Co is affected by all HMs except Ni, most extensively by the Mn salt. Br is affected by all HMs except Mn, to the greatest extent by Cu. The addition of Zn salt to the soil also increases the bioaccumulation of Na and As and very little Sr. The bioaccumulation of K, Fe, Zn, Rb, Sb, and Cs is limited, to varying degrees, using all HMs. The most significant decline of bioaccumulation factor is observed for Ca when Pb was applied to soil. As long as Ca was not determined in leaves when Mn salt was applied to soil, there are no available BC values for that treatment.

The literature is very scarce in data concerning the bioaccumulation factor or coefficient for lettuce under the impact of HM or CNT use. A significant decrease in the bioaccumulation factor of Rb, Sr, and Zn, as well as a decrease in Co, was noted when the lettuce was grown on soil fertilized with municipal solid waste compost, regardless of its application frequency. The reported data on Fe, on the contrary, show a slight increase when annual fertilization was performed and a slight decrease when biennial fertilization took place [36]. The cultivation of lettuce in a highly populated region of Nigeria, where household waste and industrial effluents were freely released into rivers that were used for irrigation [37], had led to extreme values of the bioaccumulation factor, exceeding ten times the values our experiment. The arsenic bioaccumulation factor in different lettuce cultivars was about 20 times higher than our experimental data [38]. Unfortunately, Kumwimba et al. did not evaluate the variation of elements in the lettuce other than the target element. 

### 3.2. Variation of Bioactive Compounds and Antioxidant Activity

**Figure 4:**
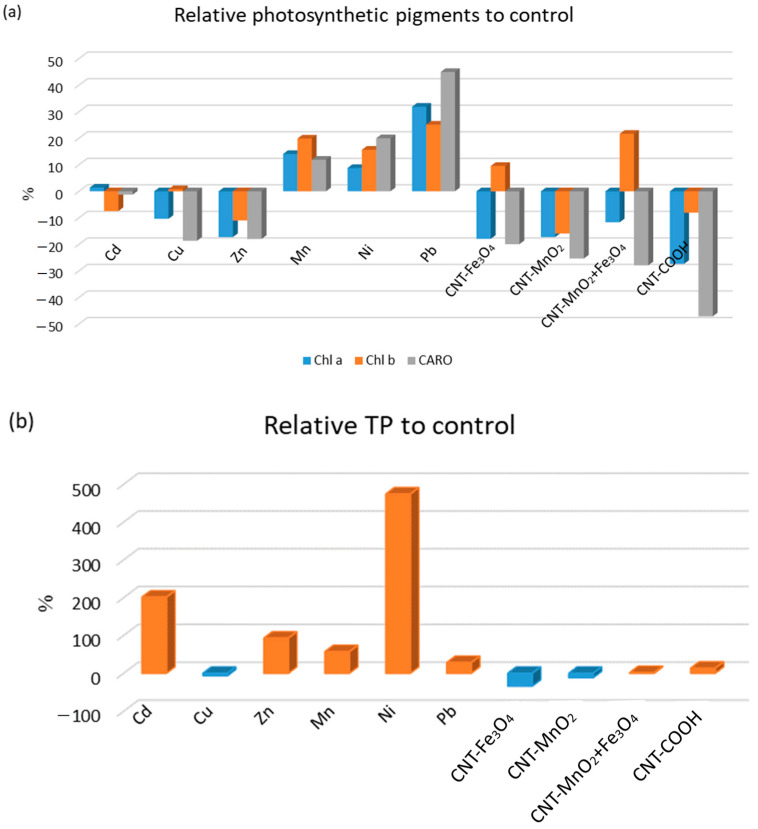

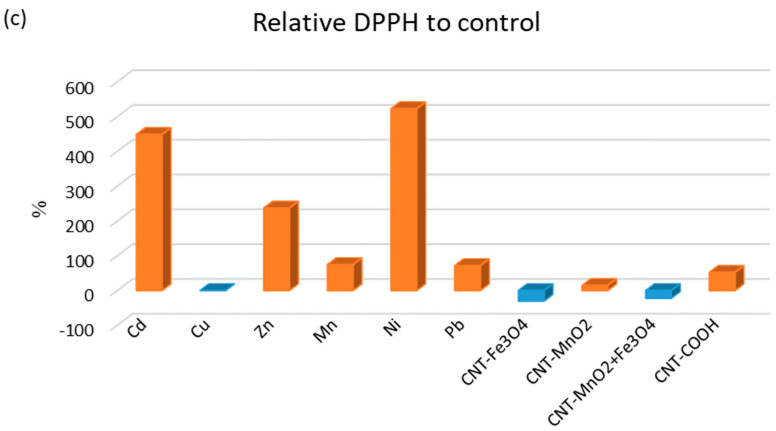
Relative values (%) of (**a**) photosynthetic pigments, (**b**) polyphenols, and (**c**) DPPH to control for lettuce grown in soils exposed to functionalized CNTs and to HMs. In (**b**,**c**), positive values are in red and negative are in blue.

The modification of bioactive compounds and antioxidant capacity was evaluated in terms of the relative variation from the control, calculated as the ratio of the difference between the value for the plants grown in the presence of HMs and functionalized CNTs and the control value multiplied by 100 and divided by the control value (Figure 4).

The response of the plant to the toxicity of Mn, Ni, Pb, Zn, CNT-MnO_2_, and CNT-COOH was the same, positive or negative, for all three parameters (chlorophyll a and b and total carotenoids), but while the use of Mn, Ni, and Pb induce pigment production, the other three treatments attenuate it. The other additives, Cu, Fe_3_O_4_-functionalized CNT, and Fe_3_O_4_-MnO_2_-functionalized CNT, increase the content of chlorophyll b while decreasing the content of chlorophyll a and total carotenoids. The use of Cd, which led to an increase in chlorophyll a and a decrease in chlorophyll b and total carotenoids content, was an exception to the aforementioned behaviors. The application of CNTs to soil modulates the antioxidant enzymes in corn seedlings, implying that CNTs can affect the antioxidant defense mechanisms [22].

The use of HM salts influenced the variation of polyphenols and antioxidant capacity to a greater extent than functionalized CNT. Among the HMs, the addition of Ni to the soil had the strongest impact, followed by Cd, Zn, Mn, and Pb, all these elements significantly increasing the values of both parameters. Cu was the only metal that caused the decrease in total polyphenol content and antioxidant capacity. Plant responses to the addition of CNT to soil varied with the functionalizing agent. The use of CNT-COOH led to higher TP and DPPH values, while CNT-Fe_3_O_4_ was responsible for their decline. The presence of CNT-MnO_2_ in soil decreased the total polyphenol content but increased the antioxidant capacity to a similar extent. An opposite influence was observed for CNT-MnO_2_Fe_3_O_4_. 

### 3.3. Correlation of the Elemental Transfer with Bioactive Compounds and Antioxidant Activity

**Figure 5:**
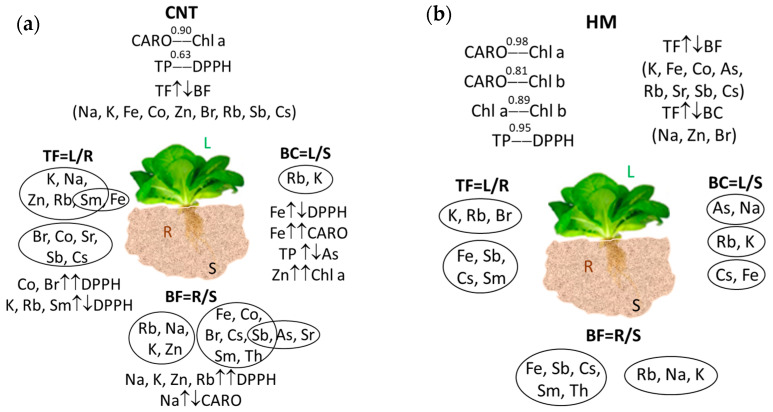
Schematic representation of the effects of functionalized CNTs (**a**) and HM salts (**b**) on lettuce. ↓↑—parameters have antagonistic behavior; ↑↑—parameters have similar behavior.

Figure 5 summarizes the most significant features that characterize the correlation of the estimated elemental transfer in terms of bioaccumulation coefficient, bioaccumulation factor, translocation factor, quantity of bioactive compounds, and antioxidant activity.

#### 3.3.1. Influence of Functionalized CNTs

The cross-correlation between bioactive compounds was generally weak when the soil was exposed to CNTs. Only carotenoid content and chlorophyll a on the one hand, and total polyphenol content and antioxidant activity on the other hand, show positive correlation, with values of 0.9 and 0.65, respectively.

The elemental uptake by root from soil was characterized by two groups of elements, within which the elements were closely correlated to each other. K, Na, Rb, and Zn formed the first group, with correlation coefficients greater than 0.76. Their translocation to the leaves was quite correlated with antioxidant activity but was slightly different to the carotenoids. The second group was the most numerous and consisted of Fe, Co, Br, Sb, Cs, Sm, and Th. As and Sr are also connected to this group, but the highest correlation was with Sb (r^2^ = 0.74).

Root-to-leaf translocation reveals two clusters of correlation. The first group consists of Br, Co, Sb, and Cs, which show correlation coefficients higher than 0.83, and Sr, whose translocation moderately correlated with the translocation of Br (r^2^ = 0.64) and less with the translocation of all other elements in the group. Translocation of Na, K, Zn, and Rb was highly correlated to each other (r^2^ > 0.88). It is interesting to note that Sm translocation correlates well with these elements (r^2^ > 0.65) but also correlates with Fe translocation, whose correlation coefficients with Na, K, Zn, and Rb do not exceed 0.5.

Soil-to-leaves transfer was characterized by only one significant correlation pair, Rb-K. On the other side, we may remark that Fe transfer was anticorrelated with antioxidant capacity and goes along with CARO. Arsenic transfer is inversely correlated to the total polyphenols content, and Zn transfer is correlated with chlorophyll a content. 

The addition of CNTs led to the anticorrelation of mineral root uptake from soil and the translocation from roots to leaves for all of elements—Na, K, Zn, and Br—proving r^2^ < −0.8. 

#### 3.3.2. Influence of HM Salts

When the soil was amended with heavy metals, the total polyphenol content and antioxidant capacity varied in a similar manner (0.95). The same thing can be said about both chlorophyll and total carotenoid concentrations (0.81–0.98). The elemental transfer from soil to leaves was neutral with respect to variation in the content of bioactive compounds, and the transfer of most elements was largely neutral with respect to the transfer of other elements. There were only three pairs of elements for which the transfer from soil to leaves was slightly correlated: Na-As, Rb-K, and Cs-Fe.

Root uptake from soil was characterized by a high mutual correlation between two groups of elements. The first group was the trinity of Na, Rb, and K, and the other consisted of Fe, Sb, Cs, Sm, and Th. Root-to-leaf K translocation was associated with Rb and Br translocation, while Fe translocation positively correlated with those of Sb, Cs, and Sm.

The addition of HMs in soil did not lead to significant correlation or antagonism between the translocation of elements from root to leaves and the bioactive compounds. As with the addition of CNTs into the soil, the supplementation with HMs was characterized by negative correlation between root uptake from soil and root-to-leaf translocations for all elements, with values of the correlation coefficient higher than −0.68. The transfer of Na, Zn, and Br to the leaves from soil and root was moderately correlated. The uptake of Na by the root was moderately anticorrelated with the translocation of K and Rb from the root to the leaves. K uptake by root was anticorrelated with As and Rb translocation from root to leaf, while its translocation from root to leaf was anticorrelated with Rb root uptake. Iron root uptake was anticorrelated with the translocation of Co, Sb, and Cs from root to leaves, and the translocation of Fe from the root to the leaves was anticorrelated with Co, Sb, and Cs root uptake. The uptake of Co by root was anticorrelated with the translocation of Sr, Sb, and Cs from the roots to the leaves. Root-to-leaf Co translocation was negatively correlated with Zn and Sb uptake by the root but positively correlated with root uptake. Root uptake of Zn and Sb was negatively correlated with the translocation of Sr and Cs from the root to the leaves, respectively.

## 4. Conclusions

The use of both groups of potential pollutants was characterized by a close correlation of K and Rb transfer to leaves from both roots and soil, as well as root uptake from soil. Root-to-leaf translocation of Sb and Cs was closely correlated regardless of the type of additive used, be they functionalized CNT or HM salts. When functionalized CNTs were applied, the number of elements showing similar translocation behavior was higher than when HM salts were used. Root uptake from soil and translocation from root to leaf appeared to be antagonistic for a large number of elements (K, Fe, Co, Rb, Sb, and Cs) suggesting that the lettuce activated the HM transfer limitation mechanism for self-protection.

The use of HM salts was characterized by the high correlation of the concentration of total carotenoid with chlorophyll b and the absence of any relevant link between elemental translocation, bioactive compounds, and antioxidant activity. On the contrary, the use of functionalized CNT showed a close correlation between antioxidant activity and root uptake of Na, K, Zn, and Rb from soil, and also root-to-leaf translocation of Co and Br.

The presence of HM salts influenced the variation of polyphenols and antioxidant capacity to a greater extent than functionalized CNT, both being much enhanced. This might mean that lettuce perceives metal salts as a more aggressive stressor than the functionalized CNTs and reacts accordingly. The effect of the additive used on the accumulation of photosynthetic pigments differed significantly from one additive to another. MnO_2_-functionalized CNT acted similarly to the Zn salt, reducing the accumulation of all types of photosynthetic pigments, while Mn, Ni, and Pb salts induced them. While the plant’s response to the presence of functionalized CNT was mainly in stimulating elemental translocation from soil to leaves relative to control plants, HM salts strongly obstructed Fe, Zn, Sb, Cs, and Sm translocation.

## Data Availability

Data are contained within the article and supplementary materials.

## Supplementary Materials

The following supporting information can be downloaded at https://www.mdpi.com/article/10.3390/metabo13121171/s1: Figure S1: Certified (CD) and experimental elemental (ED) concentration of SRM 2709 (mg/kg). * in g/kg.
