# Supplementary figures and images for: Correlation of Elemental Transfer, Bioactive Compounds and Antioxidant Activity on Lactuca sativa L. Grown in Soil with Functionalized CNT and HMs
Source: Metabolites. 2023 Nov 24;13(12):1171. doi: 10.3390/metabo13121171 (PMC10744709; doi:10.3390/metabo13121171)

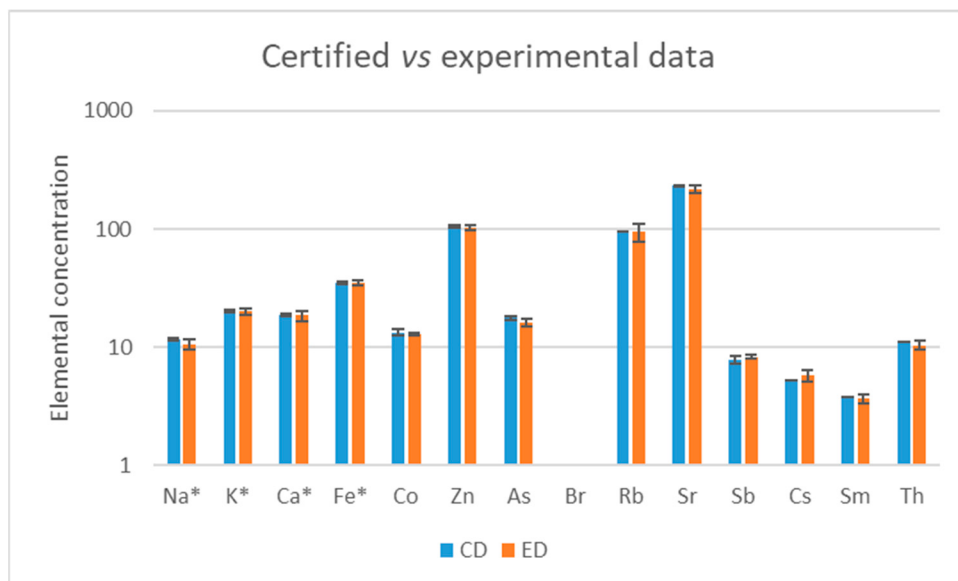

Figure S1. Certified (CD) and experimental elemental (ED) concentration of SRM 2709 (mg/kg). \* in g/kg.

Supplement: Supplementary file 1 [file metabolites-13-01171-s001.zip › metabolites-2661230-supplementary.pdf]
